# A Meta‐Analysis Reveals That the Protective Role of Silicon in Grasses Against Fungal Pathogens Depends on Infection Mechanism

**DOI:** 10.1111/pce.70586

**Published:** 2026-05-07

**Authors:** Sarah J. Thorne, Julia Cooke, Susan E. Hartley

**Affiliations:** ^1^ Plants, Photosynthesis, and Soil, School of Biosciences University of Sheffield Sheffield UK; ^2^ School of Environment, Earth and Ecosystem Science The Open University Milton Keynes UK

**Keywords:** appressorium, meta‐analysis, pathogen mode of infection, plant disease, plant‐pathogen interactions, Poaceae, silicon

## Abstract

Pathogen infection drives plant community structure and constrains global agricultural productivity. Silicon (Si) improves resistance to abiotic and herbivory stress, particularly in grasses, but relatively little attention has addressed Si‐mediated resistance to pathogens, nor has it tested how this varies according to the type of plant and pathogen and is altered by environmental factors. We performed a meta‐analysis to quantitatively assess the benefits of Si fertilisation for improving disease resistance against fungal pathogens in grasses. Overall, Si fertilisation decreased disease severity by 43% and increased biomass of infected plants by 35%. The underlying mechanisms were investigated by analysing the impacts of Si against diverse fungal infection strategies. The disease suppressive effect of Si was greater for pathogens producing appressoria, a structure involved in cell wall penetration and effector release, but lower against pathogens entering through stomata. Furthermore, Si was more effective against pathogens that produce haustoria or infection hyphae, which are involved in nutrient acquisition. These findings advance knowledge of plant‐pathogen interactions by identifying key pathogen traits determining Si effectiveness against pathogen attack in grasses. Our results support using Si fertiliser for cereal crops to reduce fungal damage and suggest Si should be considered in studies of grass evolutionary history and community structure.

## Summary statement

We performed a meta‐analysis to quantify the effect of silicon fertilisation on reducing disease severity in grasses. We found that the beneficial effect of silicon is dependent on the pathogen mode of infection and colonisation.

## 1 Introduction

Plants are challenged with numerous stresses, both abiotic and biotic, which must be minimised or overcome in order to grow and reproduce. Of the natural enemies attacking plants, pathogens are particularly diverse and damaging, especially in agricultural systems, where they are one of the most significant threats to crop production (Oerke 2006; Savary et al. 2019). In response to pathogen attack, plants divert resources away from growth to defence (M. Gao et al. 2024), and infection often induces stomatal closure, reducing photosynthesis and further reducing growth (J. Wu and Liu 2022). These impacts are reflected in average global yield losses of over 10% for rice and wheat in response to fungal and bacterial pathogen attack (Oerke 2006). Reducing the effects of pathogens on crop yield is required to ensure future food security, and thus a better understanding of how plant resistance to pathogen attack can be increased is urgently needed. Moreover, plant pathogens are a significant evolutionary and ecological influence on plants and plant community structure (Gilbert 2002) and are important in promoting both species and genotypic diversity in plant communities (Dobson and Crawley 1994; Gilbert 2002; Bever et al. 2015).

While viruses, bacteria, fungi and protists are all capable of being plant pathogens, fungal pathogens have evolved unique strategies to infect plants and have been more studied to date. Fungal pathogens use two main methods of colonisation. Firstly, they may land on the plant surface and penetrate the plant directly (Miyoshi 1895; Brown and Harvey 1927). This is commonly achieved when fungal spores germinate and produce an appressorium, which is a key infection structure. Appressoria are unicellular structures that use osmolytes such as glycerol to generate high turgor pressures, which are then channelled through hyphal structures known as penetration pegs to rupture the plant cell wall (reviewed in Ryder et al. 2022). Alternatively, some pathogens produce infection cushions to facilitate host cell wall penetration. Infection cushions are multi‐cellular structures capable of releasing large amounts of cell‐wall‐degrading enzymes (Armentrout 1987). While appressoria rely predominantly on mechanical pressure, infection cushions use a combination of high turgor pressure and enzymatic digestion to penetrate the host plant cell (Choquer et al. 2021).

Secondly, the pathogen may enter the plant via stomata or wounds (Rosen 1928; Ashworth 1935). In many species, exploratory hyphae are used to locate stomata (Knight and Sutherland 2013). Once located, either the pathogen will directly pass through the stomatal pore and enter the substomatal cavity or the pathogen will produce an appressorium‐like structure with hyphae that can enter the stomatal pore (C. Wang et al. 2011; Knight and Sutherland 2013).

The pathogen then establishes itself within the sub‐stomatal cavity (Haueisen et al. 2019). Regardless of the mode of pathogen entry, in many cases, fungal pathogen infection then involves the pathogen developing a network of invasive hyphae that spread throughout the plant (Perfect and Green 2001). Infection proceeds according to the pathogen's trophic type. Fungal pathogens can be classified as either biotrophs, hemibiotrophs or necrotrophs (Lo Presti et al. 2015). In biotrophic interactions, the pathogen obtains nutrients from living plant cells, whereas necrotrophs kill host cells prior to obtaining nutrients from them. Hemibiotrophs initially feed off living plant cells before switching to a necrotrophic lifestyle in the final stages of infection (Lo Presti et al. 2015). While such classifications are not without controversy, they nevertheless remain useful for comparing different pathogen lifestyles. For example, upon successful penetration, the penetration peg of many biotrophs differentiates into a haustorium, which is a feeding structure that facilitates nutrient uptake by creating close contact between the pathogen and plant plasma membranes (Giraldo and Valent 2013). By contrast, many necrotrophs produce infection cushions and release cell wall‐degrading enzymes (CWDEs) to digest the plant cell wall and obtain nutrients from the plant (Kubicek et al. 2014).

Fungal pathogens can also be grouped based on whether they are generalists, capable of infecting a broad range of plant species, or specialists, capable of infecting only one or a few species. While pathogens such as *Magnaporthe oryzae* overall have a broad host range, individual strains are typically highly specialised to infect a single host (Valent 1990). Such pathogens can be considered specialists. By contrast, many necrotrophs, although not all, are generalists able to infect a broad range of different plant species.

Extensive research has been dedicated to elucidating the mechanisms of plant defence against pathogens. Because the initial interaction between plants and pathogens typically occurs at the plant surface, the role of structural components is fundamental to preventing colonisation. The plant cuticle is the primary constitutive barrier against pathogen invasion (Arya et al. 2021). However, in response to cuticle penetration, plants can additionally produce induced structural defences such as localised callose deposition and lignification of the cell wall to inhibit further pathogen spread (German et al. 2022; Saini et al. 2024).

In addition to structural defences, plants also produce a variety of chemical defences to counter pathogen attack. During infection, pathogens secrete molecules known as effectors to suppress host immunity and facilitate colonisation (Lo Presti et al. 2015). In response to this, plants have evolved to detect these effectors and trigger a multi‐layered defence response. This includes the rapid accumulation of reactive oxygen species, the synthesis of antimicrobial compounds such as phytoalexins, and the expression of pathogenesis‐related proteins (Mapuranga et al. 2022).

Silicon (Si) is a beneficial element in terms of plant resistance to both abiotic and biotic stress (Debona et al. 2017; Coskun et al. 2019; Thorne et al. 2020) that is primarily deposited in the plant cell wall, in specialised silica cells in the epidermis, and on the leaf surface (Hartley et al. 2015; Zexer et al. 2023). Si deposited on the leaf surface has been shown to be an important anti‐herbivore defence, particularly against chewing herbivores (Massey and Hartley 2006, 2009; Johnson et al. 2024). The accumulation of Si in leaves can also improve plant resistance to pathogens (Rodrigues et al. 2003; Dallagnol et al. 2013; Resende, Rodrigues, Costa, et al. 2013; Resende, Rodrigues, Gomes, et al. 2013; Van Bockhaven, Spíchal, et al. 2015; Van Bockhaven, Steppe et al 2015), but the mechanisms by which Si protects against pathogen infection are less well understood, and the positive effect of Si against pathogens is not universal. For example, Si was found to be ineffective at significantly reducing disease stress in some studies (Rodgers‐Gray and Shaw 2004; Silva et al. 2010; Román Ramos et al. 2024), but the reasons for this contingency remain unclear.

Surface deposition is not the only mechanism by which Si could aid resistance to pathogen attack: during pathogen infection, Si fertilisation has been found to promote the production of phenolic compounds at sites of attempted pathogen penetration, which inhibits the progression of infection (Chérif et al. 1992; Rodrigues et al. 2003; Dorneles et al. 2018). In addition, Si has been suggested to prime the plant defence response, such that Si‐treated plants respond more quickly to attack by a pathogen (Van Bockhaven, Spíchal, et al. 2015; Van Bockhaven, Steppe, et al. 2015; Vivancos et al. 2015). Si fertilisation also has significant effects on gene expression and can activate several different defence‐related pathways, thus promoting the formation of structural barriers that inhibit pathogen penetration and increase the activity of defence‐related enzymes (Fauteux et al. 2006; M. Wang et al. 2017; L. Wang et al. 2020).

There are now a number of studies which have measured the effect of Si on disease severity in plants, which will allow a meta‐analytical approach to be applied. Recent meta‐analyses have improved our understanding about the mechanisms that underpin the beneficial effects of Si fertilisation in response to particular stresses (Cooke and Leishman 2016; Cooke and Carey 2023; F. Huang et al. 2024; Johnson et al. 2024) and this sort of quantitative analysis will allow an assessment of the factors determining the situations where Si fertilisation has a beneficial effect on plant resistance to pathogen attack, as well as the circumstances in which these benefits do not occur. It should also allow tests of the plant and pathogen traits where Si offers the most benefit, casting some light on the potential mechanisms by which Si deters pathogen attack and mitigates its impacts.

Our meta‐analysis focussed on grasses, which are high accumulators of Si, particularly in the case of major crop species, and thus are ideal candidates to investigate the mechanisms by which Si fertilisation may reduce disease stress. In addition, a focus on grasses avoids the numerous glasshouse studies on species such as lettuce and strawberry, which use foliar Si application to reduce disease stress, an artificial approach where any beneficial effects are likely to occur through different mechanisms compared to when plants accumulate Si within their tissues through plant uptake. We did include both controlled environment and field studies, anticipating that the effects of Si would likely differ in these contrasting experimental approaches, given the much greater variation in field environments. Specifically, we hypothesised that in grasses infected with fungal pathogens:

i. Si accumulation enables plants to reduce disease incidence and severity, and mitigate against biomass reduction.
ii. Si‐mediated resistance varies among grass subfamilies.
iii. The efficacy of Si varies with pathogen traits, including the mode of infection.

## 2 Materials and Methods

### 2.1 Literature Search

**Figure 1:**
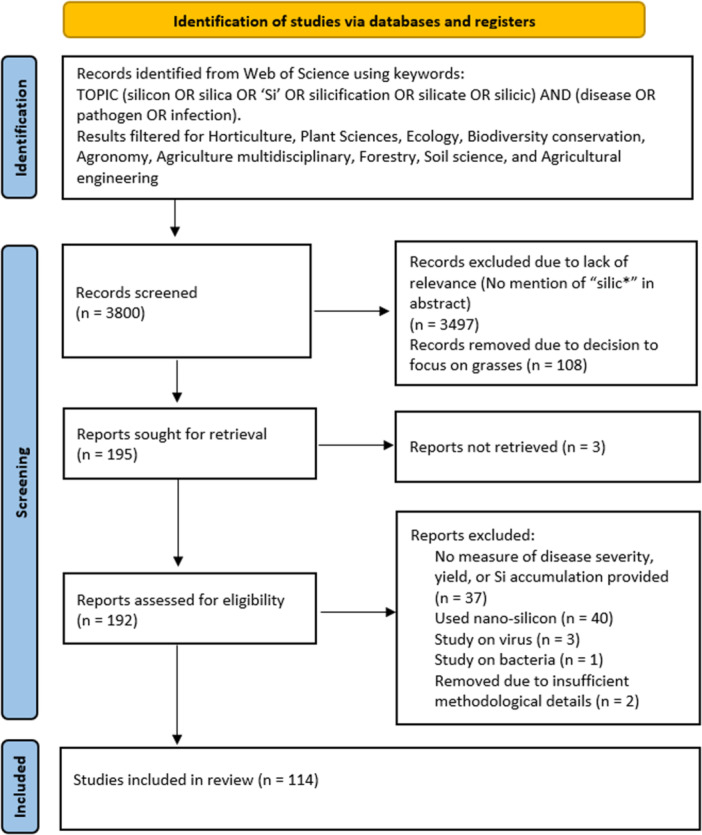
A schematic diagram of the systematic approach to identifying and collating data for analyses (adapted from PRISMA, Moher et al. 2009).

A consistent approach, based on the Preferred Reporting Items for Systematic Reviews and Meta‐analyses guidelines, was followed to systemically search, identify relevant papers and extract data (Moher et al. 2009; Koricheva and Gurevitch 2014). The Web of Science database was searched to identify papers examining the effect of Si on plants during pathogen stress using TOPIC (silicon OR silica OR ‘Si’ OR silicification OR silicate OR silicic) AND (disease OR pathogen OR infection). The results were filtered for Horticulture, Plant Sciences, Ecology, Biodiversity conservation, Agronomy, Agriculture multidisciplinary, Forestry, Soil Science and Agricultural engineering. The literature search was initially conducted on 11 March 2024 and then repeated on 9 July 2024 and 9 December 2025. A total of 3800 studies were identified, although the majority of these were epidemiology studies focused on Susceptible‐Infectious models or were not focused on pathogens. Inspection of titles and abstracts identified 286 studies of potential relevance. Selecting studies on grass species further reduced this pool (195). There were very few studies on non‐fungal pathogens; studies examining viral pathogens (two studies), elicitors (two studies), or bacteria (one study) were removed. Figure 1 demonstrates the complete literature search.

Values relating to disease severity, disease incidence, yield, growth and Si concentration were extracted. Disease incidence was defined as the prevalence of disease within a community. Disease severity was defined as any measure of the total impact of the disease on the plant. Where more than one measure of disease severity was presented, only one measure was used, with the measure selected following the hierarchy: Disease index/rating/score/severity, diseased leaf area, final lesion number, final lesion size, spore density, lesion expansion rate, relative infection efficiency, fungal development index, leaf coverage, lesion length/height/density, where disease severity was reported over multiple days, only the final day was used.

Numerical data were extracted from tables where available or extracted from plots using WebPlotDigitizer (https://automeris.io/). When presented as log or square‐root values, data were back‐transformed. All reported variances were converted to standard deviation. When Si concentration was presented as SiO_2_ concentration, values were converted to Si concentration. Forty‐four out of 114 studies provided only mean values with no variance data. For these papers, error was estimated using the average error reported across all studies. This had no significant impact on the results.

The following descriptors for plant and pathogen characteristics were extracted: (1) plant and pathogen species (where applicable, pathogen species were updated to match the current taxonomy and in instances where a pathogen has separate species names to describe the asexual and sexual stage, only one name was used), (2) study type (i.e., controlled environment or field study), (3) Si application method (i.e., foliar vs. soil application), (4) growth substrate used (i.e., hydroponics or soil). The full list of studies included is listed in the references. The pathogen infection strategy (i.e., the troph of the pathogen; the presence or absence of appressoria, infection cushions, infection hyphae, or haustorium), and whether or not a pathogen enters via the stomata, was determined by searching the literature and is indicated in Table 1.

**Table 1:** Summary of pathosystems included in the meta‐analysis. For each fungal pathogen species, host grass species and sub‐family and pathogen traits are indicated. The number of studies used in the meta‐analysis and the source of the trait information are also given.

Pathogen	Host plant	Grass sub‐family	Number of studies	Troph	Pathogen host range	Appressorium present	Haustoriumpresent	Invasive hyphae	Stomatal entry	Source
*Alternaria*	Wheat (*Triticum aestivum*)	Pooideae	1	Necrotrophic	Generalist	Yes	No	No	Yes	Thomma (2003)
Powdery mildew (*Blumeria graminis*)	Wheat (*Triticum aestivum*)	Pooideae	9	Biotroph	Specialist	Yes	Yes	No	No	Glawe (2008); Boddy (2015)
	Barley (*Hordeum vulgare*)	Pooideae	1							
	Durum wheat (*Triticum durum*)	Pooideae	1							
Dollar spot (*Clarireedia homoeocarpa*)	Creeping bentgrass (*Agrostis stolonifera*)	Pooideae	2	Necrotroph	Generalist	Infection cushion	No	No	Yes	Orshinsky et al. (2012); Sapkota et al. (2022)
Dollar spot (*Clarireedia jacksonii*)	Creeping bentgrass (*Agrostis stolonifera*)	Pooideae	1	Necrotroph	Generalist	Infection cushion	No	No	Yes	Orshinsky et al. (2012); Sapkota et al. (2022)
Leaf blight (*Cochliobolus heterostrophus*)	Maize (*Zea mays*)	Panicoideae	1	Necrotroph	Specialist	Yes	No	No	Occasionally	Yu et al. (2024)
Brown spot (Cochliobolus miyabeanus)	Rice (*Oryza sativa*)	Oryoideae	11	Necrotroph	Specialist	Yes	No	No	Occasionally	Boddy (2015); Castell‐Miller et al. (2016)
Spot blotch (*Cochliobolus sativus*)	Wheat (*Triticum aestivum*)	Pooideae	4	Necrotroph/Hemibiotroph	Generalist	Infection cushion	No	No	No	H. C. Huang and Tinline (1976); Roy et al. (2023)
	Barley (*Hordeum vulgare*)	Pooideae	2							
Leaf spot (*Cochliobolus setariae*)	Sugarcane (*Saccharum officinarum*)	Panicoideae	1	Necrotroph	Generalist	Yes	No	No	No	Roy et al. (2023)
Anthracnose (*Colletotrichum graminicola*)	Maize (*Zea mays*)	Panicoideae	1	Hemibiotroph	Specialist	Yes	No	Yes	No	Boddy (2015)
Anthracnose (*Colletotrichum sublineola*)	Sorghum bicolour	Panicoideae	7	Hemibiotroph	Specialist	Yes	No	Yes	No	Boddy (2015)
Fusarium head blight (*Fusarium culmorum*)	Wheat (*Triticum aestivum*)	Pooideae	2	Necrotroph/Hemibiotroph	Specialist	No	No	Yes	Yes	Walter et al. (2010); Torbati et al. (2021)
	Barley (*Hordeum vulgare*)	Pooideae	1							
Fusarium head blight (*Fusarium equiseti*)	Wheat (*Triticum aestivum*)	Pooideae	1	Necrotroph/Hemibiotroph	Specialist	No	No	Yes	Yes	Walter et al. (2010); Torbati et al. (2021)
	Durum wheat (*Triticum durum*)	Pooideae	1							
Fusarium head blight (*Fusarium graminearum*)	Wheat (*Triticum aestivum*)	Pooideae	3	Necrotroph/Hemibiotroph	Specialist	Infection cushion	No	Yes	Yes	Walter et al. (2010); Torbati et al. (2021)
	Durum wheat (*Triticum durum*)	Pooideae	1							
Fusarium head blight (*Fusarium solani*)	Wheat (*Triticum aestivum*)	Pooideae	1	Necrotroph/Hemibiotroph	Specialist	No	No	Yes	Yes	Walter et al. (2010); Torbati et al. (2021)
	Durum wheat (*Triticum durum*)	Pooideae	1							
Fusarium head blight (*Fusarium verticillioides*)	Wheat (*Triticum aestivum*)	Pooideae	1	Necrotroph/Hemibiotroph	Specialist	No	No	Yes	Yes	Walter et al. (2010); Torbati et al. (2021)
	Durum wheat (*Triticum durum*)	Pooideae	1							
Ring spot (*Leptosphaeria sacchari*)	Sugarcane (*Saccharum officinarum*)	Panicoideae	1	Unknown	Unknown	Unknown	Unknown	Unknown	Unknown	
Blast (Magnaporthe oryzae)	Rice (*Oryza sativa*)	Oryoideae	33	Hemibiotroph	Generalist	Yes	No	Yes	No	Boddy (2015)
	Wheat (*Triticum aestivum*)	Pooideae	8							
	Perennial ryegrass (*Lolium perenne*)	Pooideae	3							
	Finger millet (*Eleusine coracana*)	Chloridoideae	1							
	St Augustine grass (*Stenotaphrum secundatum*)	Panicoideae	1							
Leaf scald (*Monographella albescens*)	Rice (*Oryza sativa*)	Oryoideae	7	Necrotroph	Specialist	No	No	Yes	Yes	L. Araujo et al. (2016)
Eyespot (*Oculimacula yallundae*)	Wheat (*Triticum aestivum*)	Pooideae	1	Hemibiotroph	Specialist	Yes	No	Yes	No	Ramanauskienė et al. (2016)
Septoria nodorum blotch (*Parastagonospora nodorum*)	Wheat (*Triticum aestivum*)	Pooideae	2	Necrotroph	Specialist	No	No	No	No	Kariyawasam et al. (2023)
Rust (*Puccinia kuehnii*)	Sugarcane (*Saccharum officinarum*)	Panicoideae	1	Biotroph	Specialist	Yes	Yes	No	Yes	Bolton et al. (2008); Boddy (2015)
Rust (*Puccinia melanocephala*)	Sugarcane (*Saccharum officinarum*)	Panicoideae	3	Biotroph	Specialist	Yes	Yes	No	Yes	Bolton et al. (2008); Boddy (2015)
Rust (*Puccinia striiformis*)	Wheat (*Triticum aestivum*)	Pooideae	1	Biotroph	Specialist	Yes	Yes	No	Yes	Bolton et al. (2008); Boddy (2015)
Rust (*Puccinia triticina*)	Wheat (*Triticum aestivum*)	Pooideae	3	Biotroph	Specialist	Yes	Yes	No	Yes	Bolton et al. (2008); Boddy (2015)
	Durum wheat (*Triticum durum*)	Pooideae	1							
Tan spot (*Pyrenophora tritici‐repentis*)	Wheat (*Triticum aestivum*)	Pooideae	6	Necrotroph	Specialist	Yes	No	No	No	X. Liu et al. (2025)
Sheath blight/brown patch (*Rhizoctonia solani*)	Rice (*Oryza sativa*)	Oryoideae	11	Necrotroph	Generalist	Infection cushion	No	No	Yes	Molla et al. (2020)
	Creeping bentgrass (*Agrostis stolonifera*)	Pooideae	2							
	Tall fescue (*Lolium arundinaceum*)	Pooideae	1							
Southern blight (*Sclerotium rolfsii*)	Maranda grass (*Urochloa brizantha*)	Panicoideae	1	Necrotroph	Generalist	Infection cushion	No	Yes	No	Heller and Witt‐Geiges (2013); Patra et al. (2023)
Northern corn leaf blight (*Setosphaeria turcica*)	Maize (*Zea mays*)	Panicoideae	1	Hemibiotroph	Specialist	Yes	No	Yes	No	D. Wu and Turgeon (2013)
Sugarcane smut (*Sporisorium scitamineum*)	Sugarcane (*Saccharum officinarum*)	Panicoideae	1	Biotroph	Specialist	Yes	No	Yes	No	Zeng et al. (2025)
Bacterial leaf streak (*Xanthomonas translucens*)	Wheat (*Triticum aestivum*)	Pooideae	1	NA‐ bacteria	Specialist	No	No	No	Yes	Ledman et al. (2023)
Septoria leaf blotch (*Zymoseptoria tritici*)	Wheat (*Triticum aestivum*)	Pooideae	3	Necrotroph/Hemibiotroph	Specialist	No	No	Yes	Yes	Steinberg (2015)
	Durum wheat (*Triticum durum*)	Pooideae	1							

The data were checked for outliers and normality of residuals. This highlighted several outliers that fell far away from the residual line. To remove these outliers, measures with a log response ratio of greater than two or less than minus two were excluded from the analysis (82 out of 1999 measures removed). This significantly improved the model fit but had minimal impact on the results. In addition, the study by Rodgers‐Gray and Shaw (2000) was identified as having an undue influence on the model fit and test assumptions and thus was removed. This had a minimal impact on the overall conclusions. A comparison of the disease severity results with and without this study is available in Figure S1. Cultivars that were completely disease resistant were also excluded from the analysis due to generating infinite values, and because it is not possible to assess the effect of Si on cultivars that are already completely disease resistant. Funnel plots and Egger's regression tests were used to test for publication bias and did not show significant asymmetry (*t* = 0.05, *p* = 0.96).

### 2.2 Analysis

Data were analysed using R (version 4.4.1, R Core Team 2024) based on the script used in Johnson et al. (2024). The metafor package (Viechtbauer 2010) was used to calculate effect sizes, and the orchard package (Nakagawa et al. 2023) was used to visualise the results. Pairs of treatments (e.g., +Stress –Si and +Stress +Si; –Stress –Si and –Stress +Si) and one response measure (e.g., disease stress, Si, or growth) were used at a time to calculate the effect size. Hedge's D, which calculates the difference between the experimental and control group, standardised over the control group, was used to compare Si concentration. The log response ratio, which takes the logarithm of the experimental group divided by the control group, was used to assess the impact of Si addition on disease stress and growth. A phylogenetic tree for plant species included in our meta‐analytic dataset was created using the R package rotl (Michonneau et al. 2016). This tree was converted into a correlation matrix using the R package ape (Paradis and Schliep 2019), which was incorporated into subsequent meta‐analyses to control for phylogenetic dependencies.

Models were analysed using the rma.mv function in the metaphor package. Initially, data were analysed using a null model that included publication as a random factor to account for the non‐independence of results from the same study. Null model results indicated that there were significant overall effects of Si on disease severity, disease incidence, yield or biomass, and that pathogen infection significantly affected Si accumulation.

A moderator analysis was then conducted to explore factors that may influence the response to Si supplementation. This was achieved using mixed models that incorporated a combination of fixed and random effects. In these models, manuscript ID as a random factor was retained and fixed effects were plant sub‐family, pathogen mode of infection, or pathogen infection structure. In cases where a predictor could not be determined, the study was removed only for the analysis of that predictor.

In addition, environmental variables may also explain why the impact of Si on pathogen infection varies in published studies. Si may be more effective when plants are grown in controlled environments, or when plants are grown hydroponically, compared to when plants are grown in the field, due to the smaller number of confounding variables involved when using controlled environments (Poorter et al. 2016). To investigate the conditions under which Si is most effective at reducing disease severity in plants, mixed models including experimental growth conditions (field vs. controlled environment), Si source and mode of Si application were used.

Mixed‐effect model results indicated whether another factor explained a significant proportion of variation (heterogeneity) among studies. If the heterogeneity explained by the model, including a moderator (QM), was significant, the moderator was considered an important factor. The 95% confidence intervals were used to determine whether overall effect sizes for each factor were significantly different from zero or each other.

To assess whether the Si effect (i.e., the log response ratio) was correlated with the amount of Si fertiliser applied, the amount of Si applied was standardised across. For studies done hydroponically, Si application rates were converted to millimolar values based on the molecular weight of the fertiliser when application rates were provided as g/L. For studies done in soil, Si application rates were converted to tons per hectare. Where application rates were provided as g/kg, a conversion rate of 2.25 was used, which assumes fertiliser was applied to the top 15 cm of soil with a soil bulk density of 1.5 kg/L.

## 3 Results

### 3.1 Summary of Data

The meta‐analysis was conducted on 114 studies that provided 2872 observations on disease severity, disease incidence, growth and Si accumulation. These data came from 14 grass species and 31 fungal pathogen species (Table 1). When investigating the effect of Si fertilisation on disease severity, experimental conditions, pathogen mode of infection and the presence of specific pathogen infection structures were included as moderators. Investigating these moderators allowed potential mechanisms underpinning the effect of Si to be evaluated. Due to limited data, moderator analysis could not be applied to investigate the effects of pathogen stress on Si accumulation or the influence of Si on growth.

### 3.2 Si Impacts on Plant Performance and Susceptibility to Disease, Although This Is Influenced by Experimental Conditions

**Figure 2:**
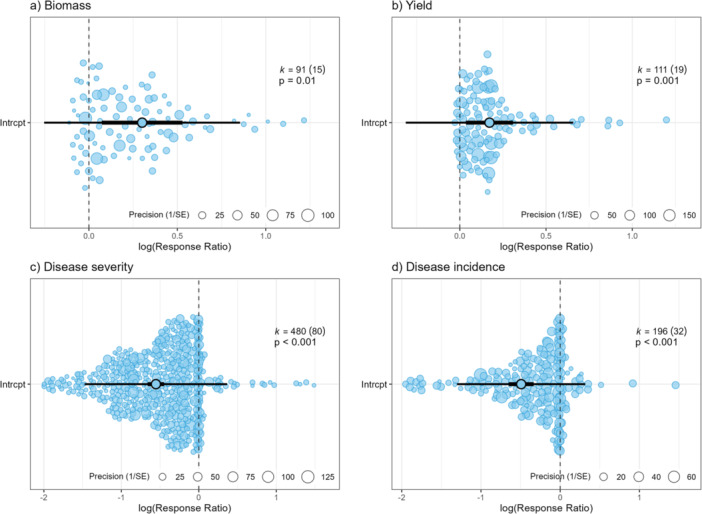
Orchard plots showing the effect of Si on (a) biomass, (b) yield, (c) disease severity and (d) disease incidence. The orchard plots show the meta‐analytic mean estimate (mean effect size, measured as log(Response Ratio)—open black circle) with its 95% confidence interval (thick horizontal whisker line) and 95% prediction interval (thin horizontal whisker line), with individual observed effect sizes as coloured circles scaled by their precision (1/SE). The number of effect sizes is denoted as *k* (with the number of studies shown in parentheses). *p* values indicate where an overall effect size is significant in a null model with publication as a random factor. [Color figure can be viewed at wileyonlinelibrary.com]

Overall, Si treatment significantly increased biomass in infected plants by 35% (*p* = 0.002, Figure 2a) and yield by 22% (*p* = 0.005, Figure 2b). Si significantly reduced disease severity by 43% (*p* < 0.001, Figure 2c), and the effect of Si on disease incidence was similar, with Si significantly reducing disease incidence by 39% (*p* < 0.001, Figure 2d). A complete forest plot showing the effect size for each study is shown in Figure S2, and Table S1 provides the complete meta‐analysis results. Eighteen studies confirmed that Si fertilisation significantly increased Si accumulation in control plants (mean effect size = 8.1, *p* < 0.001, Table S1); only fourteen studies provided measures of Si accumulation in both infected and uninfected plants, allowing the impact of infection on Si accumulation to be estimated. Pathogen infection significantly increased Si accumulation, with Si accumulation in infected plants being more than one standard deviation higher than Si accumulation in uninfected plants (mean effect size = 1.5, *p* = 0.001, Table S1).

To investigate the conditions under which Si is most effective at reducing disease severity in plants, the effect of Si in field studies was compared to that done in controlled environments. Si significantly reduced disease severity by 52% (*p* < 0.001) in controlled environment studies, but the reduction of 10% in disease severity in field studies was not statistically significant (*p* = 0.113). However, the beneficial effect of Si on disease incidence was significant in both controlled environment (45%, *p* < 0.001) and in field studies (33%, *p* < 0.001), although this effect was slightly stronger in controlled environment studies (Figure S3).

Many different Si fertilisers are currently used, including calcium and magnesium silicates, silicic acid and rice straw. A summary of the different Si sources used in the studies included in the meta‐analysis is provided in Table S2. These Si fertilisers provide differing amounts of plant‐available Si and thus may have different abilities to reduce disease severity, as evidenced by the Si source being a significant moderator. However, there was only a marginally stronger effect of Si using silicates compared to other Si sources (41% vs. 43%, respectively, *p* < 0.001, Table S1). Si fertilisers can be applied in different ways, and the Si application method was also identified as a significant moderator, although the difference in effect between foliar and root Si application was minimal (42.0% vs. 41.6%, respectively, *p* < 0.001, Table S1).

**Figure 3:**
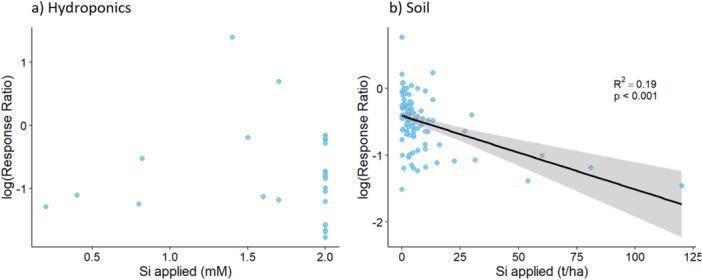
Correlation between the amount of Si applied and the effect of Si on disease severity for (a) studies where plants were grown hydroponically and (b) studies where plants were grown in soil. [Color figure can be viewed at wileyonlinelibrary.com]

To determine whether higher levels of Si fertilisation are correlated with more positive effects of Si, the amount of Si applied was standardised across studies based on whether they used hydroponics or soil. There was a positive correlation between the amount of Si applied and the suppressive effect of Si for soil‐based studies (*R* ^2^ = 0.19, *p* < 0.01, Figure 3), but not in hydroponic experiments (*R* ^2^ = 0.04, *p* = 0.868). The influence of Si on other experimental variables is listed in Table S1.

### 3.3 Si Impacts Vary Depending on Plant and Pathogen Species

**Figure 4:**
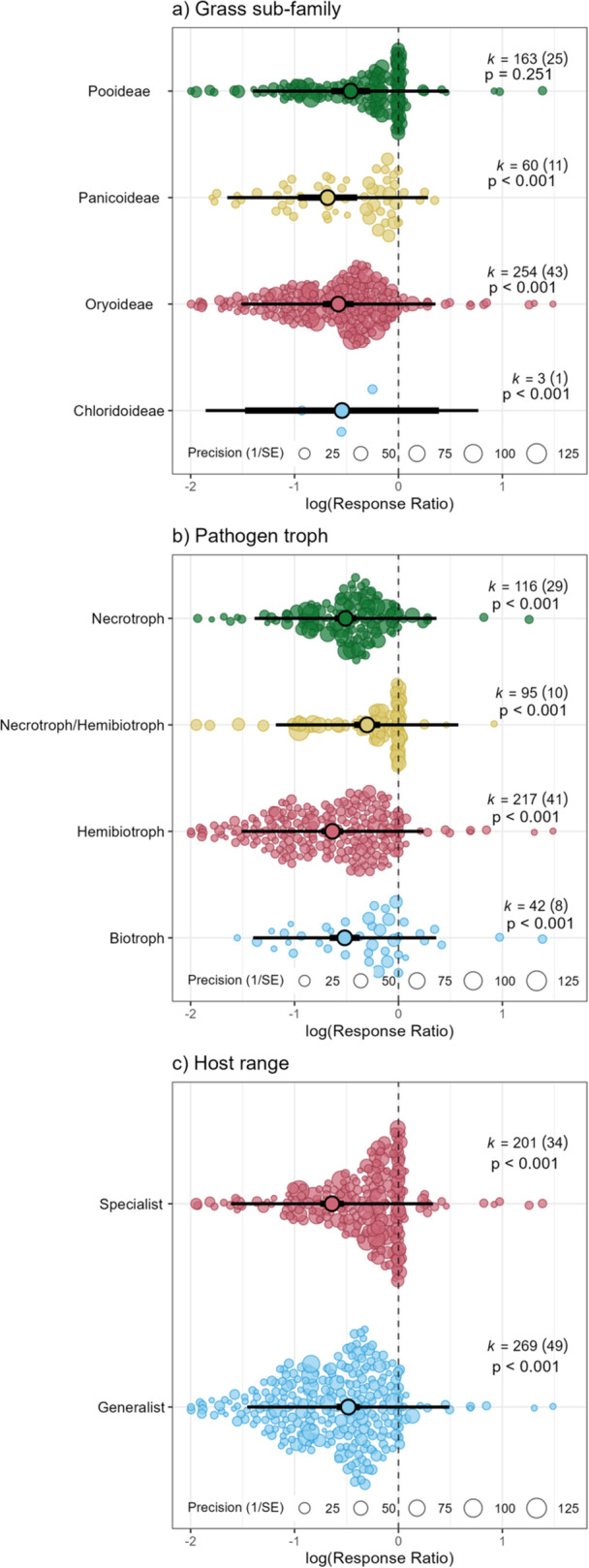
Orchard plots showing the effect of Si depending on (a) grass sub‐family, (b) pathogen troph and (c) pathogen host range on disease severity. Graphical details are explained in Figure 2. [Color figure can be viewed at wileyonlinelibrary.com]

To examine whether the effect of Si supplementation differed within the Poaceae superfamily, models were run with sub‐family as a moderator, and this addition explained significant amounts of the variation (*p* < 0.001). Si significantly reduced disease severity across all grass subfamilies apart from the chloridoideae, which were only investigated in one study. The effect of Si was slightly bigger for species in the Panicoideae (50%) compared to those in the Pooideae (37%) or Oryzoideae (44%), although this sub‐family was represented solely by rice. However, the 95% confidence intervals overlapped for all sub‐families, indicating the difference between subfamilies was not statistically significant (Figure 4a).

In addition to plant species, it was hypothesised that the effect of Si might vary among pathogens with different traits. Si significantly reduced disease severity in each of the different pathogen trophs (*p* < 0.001). There was a marginally larger effect of Si against hemibiotrophs compared to other pathogen trophs (47% reduction in disease severity for hemibiotrophs versus 40% for biotrophs versus 39% for necrotrophs, *p* < 0.001, Figure 4b). In addition, pathogen host range was a significant moderator (*p* < 0.001), with Si having a stronger effect for specialist pathogens that infect only one plant species compared to generalist pathogens that have a broad host range (48% vs. 39%, respectively, overlapping 95% CI, Figure 4c).

### 3.4 Variation in Si Impacts Depends on Pathogen Infection Strategy

Fungal pathogens vary, with key functional types defined by whether or not entry occurs through stomata, whether cell penetration structures are produced (appressorium, infection cushion) and whether or not they produce a haustorium or infection hyphae once inside the plant. Including each of these three trait groups as moderators improved the explanatory power of the model (*p* < 0.001 for all moderators, Table S1).

**Figure 5:**
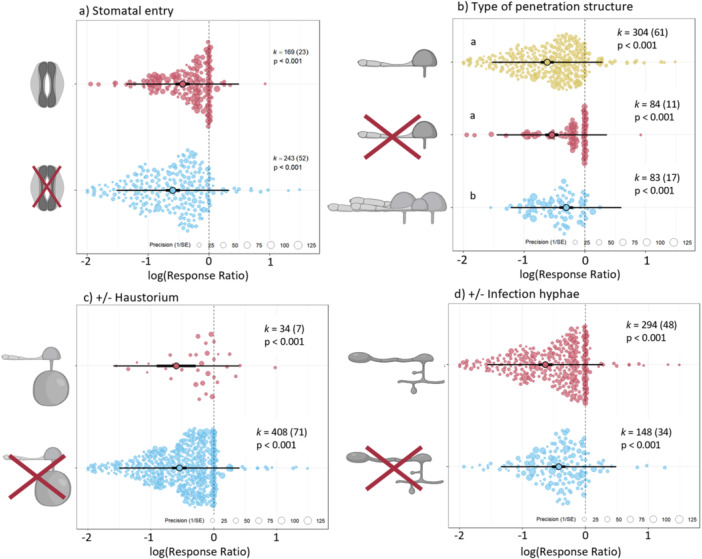
Orchard plots showing the effect of Si on disease severity based on pathogen infection strategies: (a) whether the pathogen enters the plant through the stomata, (b) type of penetration structure produced (appressorium, no specific penetration structure, or infection cushion), (c) presence of haustoria and (d) presence of infection hyphae. Graphical details are explained in Figure 2. [Color figure can be viewed at wileyonlinelibrary.com]

Si reduced disease severity by 47% against pathogens that do not enter through the stomata compared to by 38% against pathogens that do infect via the stomata (Figure 5a). Similarly, Si was significantly less effective against pathogens that produce infection cushions (26%) compared to those producing appressoria (46%) or no penetration structure (42%, Figure 5b). There was a stronger effect of Si against haustorium‐producing pathogens (45 vs. 42% haustorium vs. no haustorium, *p* < 0.001), although the only pathogens included in this study that produce haustorium were *Puccinia* species and *Blumeria graminis* (Figure 5c). Finally, the protective effect of Si was stronger against pathogens that produce infection hyphae within the plant (47%, *p* < 0.001) compared to those that do not (35%, *p* < 0.001, Figure 5d).

## 4 Discussion

### 4.1 Si Fertilisation Improves Biomass and Reduces Disease Severity in Infected Plants

Si fertilisation significantly reduced disease severity and incidence across a range of plant‐pathogen interactions, as well as positively affecting the growth and yield of infected plants. In the 14 studies where this was tested, there was a large increase in Si content in plants in response to fungal infection. Si defences are known to be upregulated in response to herbivory (Massey et al. 2007; Hartley and DeGabriel 2016), and this study suggests the same is the case for fungal pathogens, although relatively few of the studies in the meta‐analysis measured Si concentration.

The disease suppressive effect of Si fertilisation was stronger for controlled environment studies than in field studies. This is not uncommon: a similar pattern was found in a meta‐analysis examining the induction of anti‐herbivore defence traits (Ojha et al. 2022). The more variable environment in the field, where plants experience a range of other biotic and abiotic stresses, potentially makes the impact of pathogens on plant performance harder to discern, and it is likely that there are greater and less variable disease impacts in enclosed spaces. In addition, many controlled environment studies, particularly those conducted hydroponically, involve growing plants in artificially low levels of Si compared to the soil Si availability in field studies, so Si fertilisation produces a similarly artificial large increase in uptake and associated benefits in these cases.

Overall, the efficacy of different Si fertilisers and methods of application was similar. However, there was a weak correlation between the amount of Si applied and the reduction in disease severity due to Si (Figure 3), although this was only the case for plants grown in soil and not found in those grown hydroponically. Si application does not necessarily correlate directly with Si availability to plants; for example, pH and soil type strongly influence plant Si availability in the soil (Gocke et al. 2013). There is more plant‐available Si when plants are grown hydroponically, and thus hydroponic plants often have a surplus of Si, reducing the likelihood of observing a correlation between the additional Si applied and a positive Si effect.

### 4.2 The Effect of Si Fertilisation Is Dependent Upon Grass Subfamily and Pathogen Troph

Si fertilisation had a positive effect in reducing disease severity across an array of grass crop species, and the positive effect of Si fertilisation was found across three of the four sub‐families spanning the two main grass clades. This effect was largest in the Panicoideae, which is mostly comprised of species that occur in warm temperate and tropical regions. Plants from these regions may have evolved greater resilience to pathogens, given that most plant diseases are favoured by high rainfall, air humidity and soil moisture (Velásquez et al. 2018). However, a significant proportion of these experimental studies focussed on rice blast (26 out of 82 studies), and the effect of Si on many economically important pathogens, such as *Zymoseptoria tritici* and *Mycosarcoma maydis*, has not yet been thoroughly examined (Dean et al. 2012).

Si fertilisation was effective against a broad range of pathogens, but particularly those with high host specificity rather than generalists (Figure 4). It is hypothesised that the broad protective effect of silicification, which incorporates both physical defences and the potential priming of biochemical defence pathways, may be a more complex evolutionary hurdle for pathogens to overcome compared to the circumvention of single‐locus, gene‐for‐gene immunity (Van Bockhaven et al. 2013). Our results contradict the hypothesis that Si is ineffective against necrotrophic pathogens (Coskun et al. 2019), although Si was less effective against pathogens that infect using infection cushions, which are structures commonly produced by necrotrophic pathogens and produce a larger array of CWDEs compared to unicellular appressoria (Choquer et al. 2021).

### 4.3 Si Fertilisation Is More Effective Against Pathogens That Penetrate the Cell Wall

**Figure 6:**
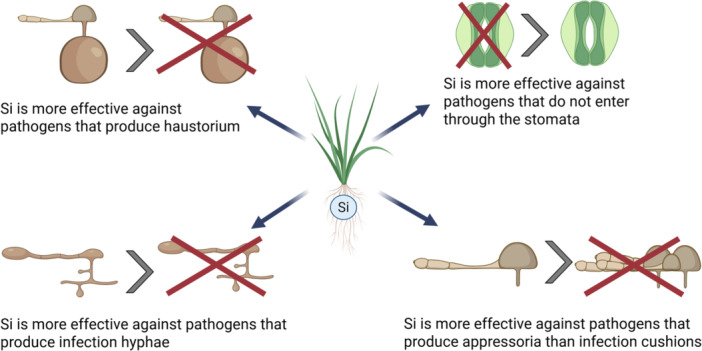
Diagram showing key findings from the meta‐analysis. Variation in the effect of Si with the mode of entry or the infection structures produced by the pathogen offers insights into the possible mechanisms underpinning the beneficial effects of Si. Figure created using Biorender (https://www.biorender.com). [Color figure can be viewed at wileyonlinelibrary.com]

Si fertilisation was more effective against pathogens that do not enter the plant through stomata (Figure 5) but use mechanisms to penetrate the cell surface. Si fertilisation is associated with increased phenolic deposition at sites of attempted penetration by pathogens (Rémus‐Borel et al. 2005; G. Zhang et al. 2013). The accumulation of phenolics promoted by Si can inhibit the proper formation of haustoria and development of infection hyphae (Bélanger et al. 2003; Dorneles et al. 2018), which may explain why in this meta‐analysis, Si was found to be more effective against pathogens that produce these infection structures. Furthermore, Si can inhibit the release of effectors from infection structures, including appressoria and haustoria, potentially explaining the larger beneficial effect of Si against pathogens that produce these infection structures (Rasoolizadeh et al. 2018, 2020). The way Si is deposited on and around the plant surface is known to affect the resistance of plants to herbivores: herbivore guilds able to bypass Si deposits are less impacted compared to those that consume the leaf surface directly (Massey et al. 2006; Johnson et al. 2024). Whether these sorts of mechanisms determine the differential effects on pathogens with different infection structures observed here is unknown, as are the patterns of Si deposition in response to attach by different pathogen types (Figure 6).

### 4.4 Future Work and Implications for Sustainable Agriculture

The Poaceae dominate the study of Si in the interactions between plants and their natural enemies, as this family is the largest group of high Si‐accumulating plant species (Hodson et al. 2005; Deshmukh et al. 2020). Many studies of the beneficial effects of Si in plant resistance to herbivory have been in natural ecosystems (Cooke and Leishman 2011; Hartley and DeGabriel 2016), but, although pathogens are known to significantly influence grassland communities (Mitchell 2003; Allan et al. 2010), the studies with data suitable for inclusion in this meta‐analysis were largely from agriculture. The lack of studies of the benefits of Si accumulation for plants under pathogen attack in non‐agricultural systems is a significant knowledge gap.

The most important crops globally are Si‐accumulating grasses, and Si fertiliser is increasingly being applied to crops to improve growth and resistance to both abiotic and biotic stress (Debona et al. 2017; Coskun et al. 2019; Thorne et al. 2020). This analysis revealed consistent increases in biomass and reductions in disease incidence with Si addition to plants exposed to pathogens, irrespective of pathogen or plant species, which shows promise for Si application as an alternative to fungicides for protecting crops against disease with much lower negative environmental impacts. These results also showed that the specific type of Si fertiliser used, and whether foliar or soil application was used, have only marginal overall effects on the efficacy of Si fertilisation. However, for soil studies, higher levels of Si fertilisation are correlated with greater reductions in disease severity. Finally, the results highlight the contrasting impacts of Si on fungal pathogens which use different infection mechanisms. This should be explored further in future work, particularly in non‐model pathosystems, but it is clear from these findings that some pathogens are more impacted by Si application than others. That could inform more targeted use of Si fertiliser in sustainable agriculture by directing applications towards pathogens particularly vulnerable to Si defences.

## Conflicts of Interest

The authors declare no conflicts of interest.

## Supporting information

Supporting File 1

## Data Availability

The data that support the findings of this study are openly available in Dryad at https://doi.org/10.5061/dryad.gmsbcc31c.
