# Supplementary material for: A Meta‐Analysis Reveals That the Protective Role of Silicon in Grasses Against Fungal Pathogens Depends on Infection Mechanism
Source: Plant Cell Environ. 2026 May 7;49(8):5947–64. doi: 10.1111/pce.70586 (PMC13353730; doi:10.1111/pce.70586)
Supplement: Supplementary file 1 — Supporting File 1 [file PCE-49-5947-s001.docx]

## Supplementary Information


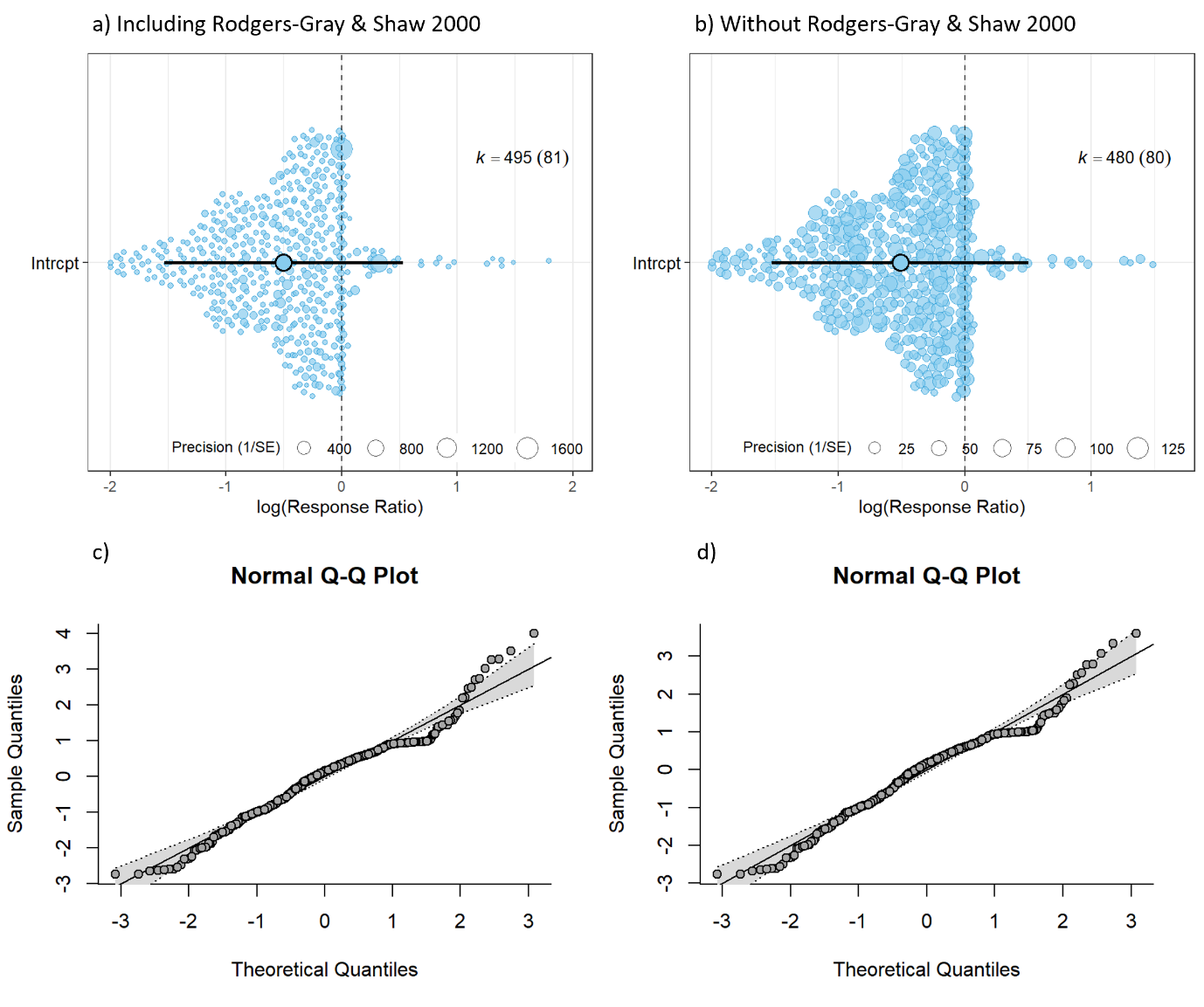


Supplementary Figure 1: a) and b) Comparison of disease severity results and c) and d) q-q plots with (a and c) and without (b and d) the influential study Rodgers-Gray and Shaw 2000.


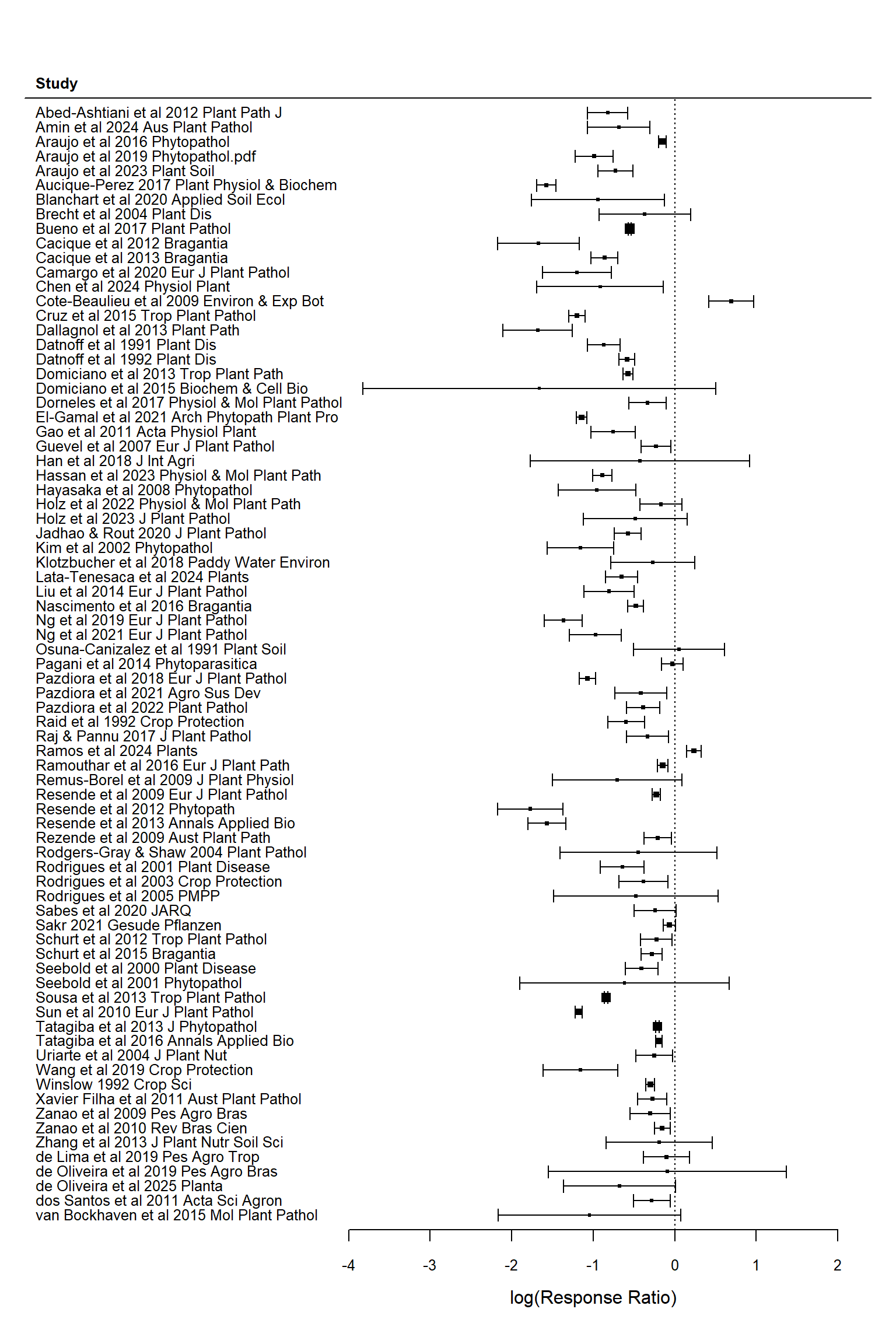


Supplementary Figure 2: Forest plot showing the average response ratio (small black points) for each study for the effect of Si on disease severity. Error bars show the 95% confidence interval.


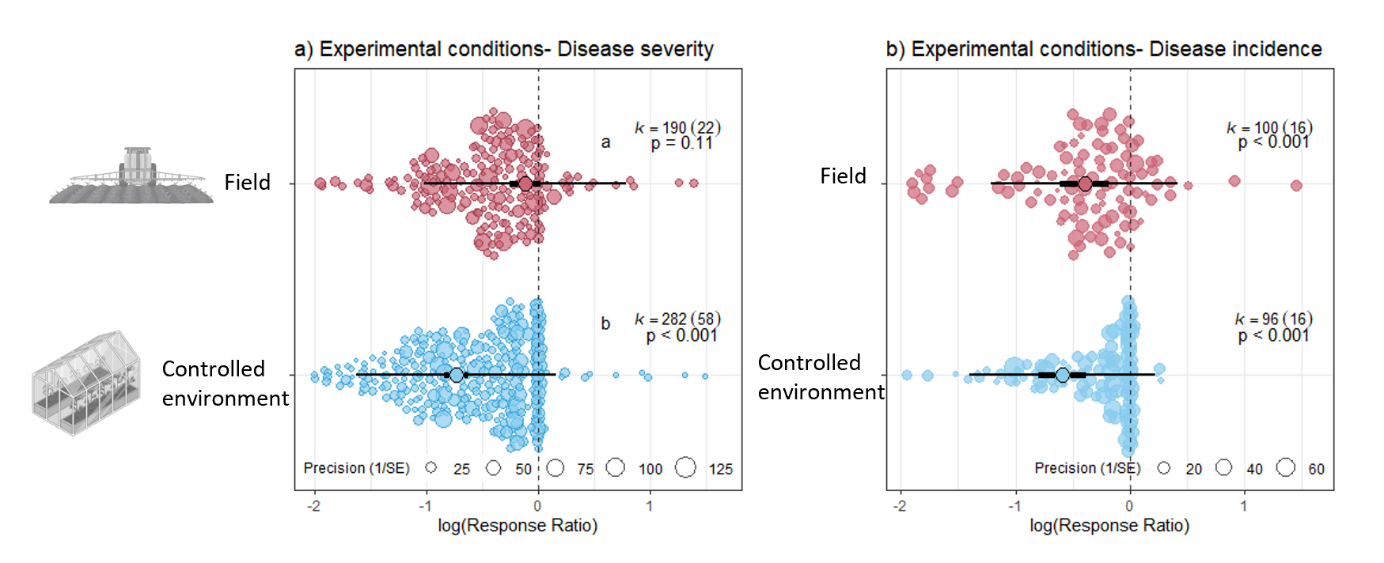


Supplementary Figure 3: Orchard plot showing the effect of Si depending on environmental conditions for a) Disease severity and b) Disease incidence. Graphical details are explained in Figure 2.

Supplementary Table 1: Complete list of meta-analysis results. In each row, the null models (no fixed factor) are reported with manuscript ID included as a random factor. The number of data points in each analysis is given (*k*) and *Q*_E_ is the amount of unexplained heterogeneity. Subsequent models with plant species (wheat/rice/other), experimental conditions (field/controlled environment), growth substrate (soil/hydroponics), Si source (silicate/not silicate), Si application method (foliar/root), days post infection (7/14 dpi), stomatal entry (yes/no), host range (generalist/specialist), pathogen troph (necrotroph/biotroph/hemibiotroph) and presence/absence of appressorium, infection hyphae, or haustorium are reported, and *Q*_M_ is indicative of the heterogeneity explained by the structured model, with a significant *p*-value showing the amount of variation explained that is more than the null model.

| **Response** | **Fixed factors** | ***k*** | **df** | **AIC** | ***Q*_E_** | ***p*** | ***Q*_M_** | ***p*** |
| --- | --- | --- | --- | --- | --- | --- | --- | --- |
| Disease severity | NA | 480 | 479 | 20846 | 41353 | < 0.001 |  |  |
|  | Grass subfamily | 452 | 448 | 19845 | 39807 | < 0.001 | 97.4 | < 0.001 |
|  | Experimental conditions | 444 | 442 | 19667 | 39172 | < 0.001 | 192.7 | < 0.001 |
|  | Growth substrate | 268 | 266 | 7311 | 19419 | < 0.00.1 | 96.6 | < 0.001 |
|  | Si source | 452 | 450 | 19847 | 38485 | < 0.001 | 100.0 | < 0.001 |
|  | Si application method | 450 | 448 | 19686 | 39921 | < 0.001 | 96.0 | < 0.001 |
|  | Dpi (7 vs 14 dpi) | 316 | 314 | 1355 | 4207 | < 0.001 | 107.5 | < 0.001 |
|  | Stomatal entry | 412 | 410 | 13261 | 31098 | < 0.001 | 184.7 | < 0.001 |
|  | Host range | 442 | 440 | 18606 | 36694 | < 0.001 | 373.5 | < 0.001 |
|  | Appressorium | 442 | 439 | 18281 | 38909 | < 0.001 | 718.2 | < 0.001 |
|  | Infection hyphae | 442 | 440 | 18407 | 39177 | < 0.001 | 585.3 | < 0.001 |
|  | Pathogen troph | 442 | 438 | 18814 | 37596 | < 0.001 | 184.8 | < 0.001 |
|  | Haustorium | 442 | 440 | 18888 | 39087 | < 0.001 | 92.8 | < 0.001 |
| Disease incidence | NA | 196 | 195 | 13453 | 19912 | < 0.001 |  |  |
|  | Experimental conditions | 196 | 194 | 13451 | 19788 | < 0.001 | 39.8 | < 0.001 |
| Biomass- uninfected plants | NA | 23 | 22 | 42 | 157.8 | < 0.001 |  |  |
| Biomass- infected plants | NA | 91 | 90 | 2074 | 2998.9 | < 0.001 |  |  |
| Yield | NA | 111 | 110 | 2539 | 5079.2 | < 0.001 |  |  |
| Si | NA | 19 | 18 | 109 | 98.6 | < 0.001 |  |  |

Supplementary Table 2: List of studies included in the meta-analysis. Response measures recorded are indicated.

| Study | Disease severity | Disease incidence | Biomass or Yield | Si |
| --- | --- | --- | --- | --- |
| Abed-Ashtiani et al 2012 Plant Path J | Yes | Yes | Yes |  |
| Araujo et al 2016 Phytopathol | Yes |  |  |  |
| Araujo et al 2019 Phytopathol.pdf | Yes |  |  | Yes |
| Araujo et al 2023 Plant Soil | Yes |  |  | Yes |
| Aucique-Perez 2017 Plant Physiol & Biochem | Yes |  |  | Yes |
| Brecht et al 2004 Plant Dis | Yes |  |  |  |
| Buck et al 2008 J Plant Nut |  | Yes |  |  |
| Bueno et al 2017 Plant Pathol | Yes |  |  |  |
| Cai et al 2008 Physiol Plant |  | Yes |  | Yes |
| Camargo et al 2020 Eur J Plant Pathol | Yes |  |  |  |
| Chen et al 2024 Physiol Plant | Yes | Yes |  |  |
| Cote-Beaulieu et al 2009 Environ & Exp Bot | Yes |  |  |  |
| Cruz et al 2015 Trop Plant Pathol | Yes |  |  |  |
| Dallagnol et al 2009 Phytopath |  | Yes |  |  |
| Dallagnol et al 2013 Plant Path | Yes |  | Yes |  |
| Dallagnol et al 2023 Trop Plant Pathol.pdf |  |  | Yes |  |
| Datnoff et al 1991 Plant Dis | Yes | Yes | Yes |  |
| Datnoff et al 1992 Plant Dis | Yes | Yes | Yes |  |
| Deng et al 2020 FIPS |  | Yes |  |  |
| Domiciano et al 2010 J Phytopathol | Yes |  |  |  |
| Domiciano et al 2010 Trop Plant Pathol | Yes |  |  |  |
| Domiciano et al 2013 Trop Plant Path | Yes |  |  |  |
| Domiciano et al 2015 Biochem & Cell Bio | Yes |  |  |  |
| Dorneles et al 2017 Physiol & Mol Plant Pathol | Yes |  |  |  |
| Dorneles et al 2018 Planth Pathol | Yes |  |  |  |
| dos Santos et al 2011 Acta Sci Agron | Yes | Yes |  |  |
| dos Santos et al 2014 Revista Caatinga | Yes | Yes |  |  |
| El-Gamal et al 2021 Arch Phytopath Plant Pro | Yes |  | Yes |  |
| Gao et al 2011 Acta Physiol Plant | Yes |  |  | Yes |
| Guevel et al 2007 Eur J Plant Pathol | Yes |  | Yes |  |
| Hassan et al 2023 Physiol & Mol Plant Path | Yes |  |  |  |
| Hayasaka et al 2008 Phytopathol | Yes |  |  |  |
| Holz et al 2022 Physiol & Mol Plant Path | Yes |  |  |  |
| Holz et al 2023 J Plant Pathol | Yes |  |  |  |
| Jadhao & Rout 2020 J Plant Pathol | Yes |  | Yes |  |
| Junior et al 2009 J Phytopathol |  | Yes |  |  |
| Kim et al 2002 Phytopathol | Yes |  |  |  |
| Klotzbucher et al 2018 Paddy Water Environ | Yes |  | Yes |  |
| Lata-Tenesaca et al 2024 Plants | Yes |  |  | Yes |
| Liu et al 2014 Eur J Plant Pathol | Yes | Yes |  |  |
| Mochko et al 2024 Plant Soil | Yes |  |  | Yes |
| Moldes et al 2016 Acta Physiol Plant |  |  | Yes |  |
| Nakata et al 2008 Crop Protection |  | Yes |  |  |
| Nanayakkara et al 2008 Plant Dis | Yes | Yes |  |  |
| Nascimento et al 2018 Rev Ca | Yes |  | Yes |  |
| Naz et al 2021 Plant Physiol & Biochem |  | Yes | Yes |  |
| Ng et al 2019 Eur J Plant Pathol | Yes |  |  | Yes |
| Ng et al 2021 Eur J Plant Pathol | Yes |  |  |  |
| Nicchio et al 2024 J Plant Nut |  |  | Yes |  |
| Osuna-Canizalez et al 1991 Plant Soil | Yes |  |  |  |
| Pagani et al 2014 Phytoparasitica | Yes | Yes |  |  |
| Pazdiora et al 2018 Eur J Plant Pathol | Yes |  |  |  |
| Pazdiora et al 2021 Agro Sus Dev | Yes | Yes | Yes |  |
| Pazdiora et al 2022 Plant Pathol | Yes | Yes |  |  |
| Pereira et al 2020 Theor Exp Plant Physiol.pdf |  |  |  | Yes |
| Rahman et al 2015 Phytopathol | Yes | Yes |  |  |
| Raid et al 1992 Crop Protection | Yes |  |  |  |
| Ramos et al 2024 Plants | Yes |  |  |  |
| Ramouthar et al 2016 Eur J Plant Path | Yes |  |  |  |
| Remus-Borel et al 2009 J Plant Physiol | Yes |  |  |  |
| Resende et al 2009 Eur J Plant Pathol | Yes |  |  |  |
| Resende et al 2012 Phytopath | Yes |  |  |  |
| Resende et al 2013 J Phytopathol |  |  | Yes |  |
| Rezende et al 2009 Aust Plant Path | Yes | Yes |  |  |
| Rodgers-Gray & Shaw 2004 Plant Pathol | Yes | Yes |  |  |
| Rodrigues et al 2001 Plant Disease | Yes |  | Yes |  |
| Rodrigues et al 2003 Crop Protection | Yes |  |  |  |
| Rodrigues et al 2005 PMPP | Yes |  |  |  |
| Sakr & Kurdali 2023 Gesunde Pflanzen | Yes | Yes |  |  |
| Sakr 2021 Gesude Pflanzen | Yes | Yes |  |  |
| Schmidt et al 1999 J Plant Nut |  | Yes |  |  |
| Schurt et al 2012 Trop Plant Pathol | Yes |  |  |  |
| Seebold et al 2000 Plant Disease | Yes |  | Yes |  |
| Seebold et al 2001 Phytopathol | Yes |  |  |  |
| Seebold et al 2004 Plant Disease.pdf |  | Yes | Yes |  |
| Sester et al 2019 Crop Protection | Yes |  | Yes |  |
| Silveira et al 2021 J Pythopath |  |  |  | Yes |
| Sousa et al 2013 Trop Plant Pathol | Yes | Yes |  |  |
| Sun et al 2010 Eur J Plant Pathol | Yes | Yes | Yes | Yes |
| Tatagiba et al 2013 J Phytopathol | Yes |  |  |  |
| Telaxka et al 2019 Rev Bras Cienc Agrar.pdf | Yes | Yes | Yes |  |
| Trebbi et al 2021 Agriculture | Yes |  | Yes |  |
| van Bockhaven et al 2015 Mol Plant Pathol | Yes |  |  |  |
| Wang et al 2019 Crop Protection | Yes |  |  |  |
| Wiese et al 2005 J Plant Nut Soil Sci |  | Yes |  |  |
| Winslow 1992 Crop Sci | Yes |  | Yes |  |
| Wu et al 2015 Aust Plant Pathol | Yes |  |  |  |
| Xavier Filha et al 2011 Aust Plant Pathol | Yes |  |  |  |
| Zhang et al 2013 J Plant Nutr Soil Sci | Yes |  |  |  |
| Han et al 2018 J Int Agri | Yes | Yes | Yes |  |
| Zanao et al 2009 Pes Agro Bras | Yes |  | Yes |  |
| de Lima et al 2019 Pes Agro Trop | Yes |  |  |  |
| Zanao et al 2010 Rev Bras Cien | Yes |  |  |  |
| Sipahutar et al 2025 Cien Agro |  | Yes | Yes |  |
| Guimaraes et al 2020 Rec FCA |  | Yes |  |  |
| Blanchart et al 2020 Applied Soil Ecol | Yes |  | Yes |  |
| Cacique et al 2013 Bragantia | Yes |  |  |  |
| Uriarte et al 2004 J Plant Nut | Yes | Yes |  |  |
| Wani et al 2024 Plant Physiol & Biochem |  |  |  | Yes |
| de Curtis et al 2012 Field Crop Res |  |  | Yes |  |
| Sabes et al 2020 JARQ | Yes | Yes | Yes |  |
| Zhang et al 2006 Crop Sci |  | Yes |  |  |
| Resende et al 2013 Annals Applied Bio | Yes |  |  |  |
| Amin et al 2024 Aus Plant Pathol | Yes | Yes | Yes |  |
| Nascimento et al 2016 Bragantia | Yes |  |  |  |
| Schurt et al 2015 Bragantia | Yes |  |  |  |
| Santos et al 2014 Rev Cien Agro | Yes |  |  |  |
| Leusch & Buchenauer 1989 Zeitschrift |  | Yes | Yes |  |
| Tatagiba et al 2016 Annals Applied Bio | Yes |  |  | Yes |
| de Oliveira et al 2025 Planta | Yes |  | Yes |  |
| Cacique et al 2012 Bragantia | Yes |  |  |  |
| de Oliveira et al 2019 Pes Agro Bras | Yes |  | Yes |  |
| Raj & Pannu 2017 J Plant Pathol | Yes |  | Yes |  |
| Provance-Bowley et al 2010 Soil Science Soc of Amer J |  |  | Yes |  |
